# COVID-19 has heightened tensions between and exposed threats to core values of emergency medicine

**DOI:** 10.1007/s43678-022-00383-0

**Published:** 2022-09-10

**Authors:** Eve Purdy, Gillian Forster, Hayley Manlove, Laura McDonough, Meredith Powell, Krista Wood, Louise Rang, Damon Dagnone, Rob Brison, Doug Henry, Stuart L. Douglas

**Affiliations:** 1 Department of Emergency Medicine, Gold Coast University Hospital, Southport, QLD Australia; 2 Department of Emergency Medicine, Queen’s University, Kingston, Canada; 3 Emergency Department, Kingston Health Sciences Centre, Kingston, Canada; 4 School of Medicine, Queen’s University, Kingston, Canada; 5 Department of Anthropology, University of North Texas, Denton, USA; 6 Department of Critical Care Medicine, Queen’s University, Kingston, Canada; 7 Translational Simulation Collaborative, Bond University, Gold Coast, Australia

**Keywords:** Emergency medicine, Ethnography, COVID-19, COVID-19, Ethnographie, Recherche qualitative

## Abstract

**Background:**

Professional culture is a powerful influence in emergency departments, but incompletely understood. Disasters magnify cultural realities, and as such the COVID-19 pandemic offered a unique opportunity to better understand emergency medicine (EM) values, practices, and beliefs.

**Methods:**

We conducted a collaborative ethnography at a tertiary care center during the acute phase of the response to the threat of COVID-19 (March–May 2020). Collaborative ethnography is a method that partners directly with communities during design, data gathering, and analysis to study culture. An ED-based research team gathered data including field notes from 300 h of participant observation and informal interviews, 42 semi-structured interviews, and 57 departmental documents. Data were deductively coded using a previously generated framework for understanding EM culture.

**Results:**

Each of seven core values from the original framework were identified in the dataset and further contextualized understanding of EM culture. COVID-19 exacerbated pre-existing tensions and threats to the core values of EM. For example, the desire to provide patient-centered care was impeded by strict visitor restrictions; the ability to treat life-threatening illness was impaired by new resuscitation room layouts and infection control procedures; and subtle changes in protocols had downstream impact on flow and the ability to balance needs and resources at a system level. The cultural values related to teams were protective and strengthened during this time. The pandemic exposed problems with the status quo, underscored inherent tensions between ED values, and highlighted threats to self-identity.

**Conclusion:**

COVID-19 has highlighted and compounded existing tensions and threats to the core values of EM, underscoring a critical mismatch between values and practice. Realignment of the realities of ED work with staff values is urgently needed.

**Supplementary Information:**

The online version contains supplementary material available at 10.1007/s43678-022-00383-0.

## Résumé

**Contexte:**

La culture professionnelle est une influence puissante dans les services d'urgence, mais elle est incomplètement comprise. Les catastrophes amplifient les réalités culturelles et, à ce titre, la pandémie de COVID-19 a offert une occasion unique de mieux comprendre les valeurs, les pratiques et les croyances de la médecine d'urgence (MU).

**Méthodes:**

Nous avons mené une ethnographie collaborative dans un centre de soins tertiaires pendant la phase aiguë de la réponse à la menace du COVID-19 (mars-mai 2020). L’ethnographie collaborative est une méthode qui s’associe directement aux communautés pendant la conception, la collecte de données et l’analyse pour étudier la culture. Une équipe de recherche basée à l'urgence a recueilli des données, y compris des notes de terrain tirées de 300 heures d’observation des participants et d’entrevues informelles, de 42 entrevues semi-structurées et de 57 documents ministériels. Les données ont été codées de manière déductive à l'aide d'un cadre précédemment créé pour comprendre la culture de la MU.

**Résultats:**

Chacune des sept valeurs fondamentales du cadre original a été identifiée dans l'ensemble de données et a permis de mieux comprendre la culture de la MU. COVID-19 a exacerbé les tensions préexistantes et les menaces qui pèsent sur les valeurs fondamentales de la MU. Par exemple, le désir de fournir des soins centrés sur le patient a été entravé par des restrictions strictes concernant les visiteurs ; la capacité de traiter des maladies potentiellement mortelles a été compromise par les nouvelles dispositions des salles de réanimation et des procédures de contrôle des infections ; et des changements subtils dans les protocoles ont eu un impact en aval sur le flux et la capacité à équilibrer les besoins et les ressources au niveau du système. Les valeurs culturelles liées aux équipes étaient protectrices et renforcées pendant cette période. La pandémie a mis en évidence les problèmes liés au statu quo, souligné les tensions inhérentes entre les valeurs des services d’urgence et mis en évidence les menaces pour l'identité personnelle.

**Conclusion:**

COVID-19 a mis en évidence et aggravé les tensions et les menaces existantes pour les valeurs fondamentales de la MU, soulignant un décalage critique entre les valeurs et la pratique. Il est urgent de réaligner les réalités du travail dans les services d'urgence sur les valeurs du personnel.

## Clinician’s capsule

**Table:** 

***What is known about the topic?***
Professional culture is important to the practice of emergency medicine (EM) but incompletely understood. Disasters are a time when culture can be easily studied, as such COVID-19 presented an opportunity to learn more about the culture of EM.
***What did this study ask?***
How does COVID-19 expose and challenge our collective EM values and beliefs?
***What did this study find?***
There are tensions between, and threats to the values central to EM that were exposed by COVID-19. The values and beliefs that clinicians hold do not always align with the realities of the practice.
***Why does this study matter to clinicians?***
The tensions between values and practice provides an explanation for understanding the challenges emergency clinicians face and provides impetus for organizations to align operations with what matters most to clinicians.

## Introduction

Professional culture—the values, beliefs and practices that underpin a group—is a powerful influence in emergency departments (EDs), but incompletely understood [1–5]. Although emergency clinicians often draw strength from their professional culture, they can also become distressed when workplace realities conflict with their emergency medicine (EM) values and beliefs [6–8]. A deeper understanding of EM culture might help clinicians recognize values and beliefs as a source of support in their work, and also to help understand growing unease in the face of escalation of challenging workplace realities.

**Fig. 1:**
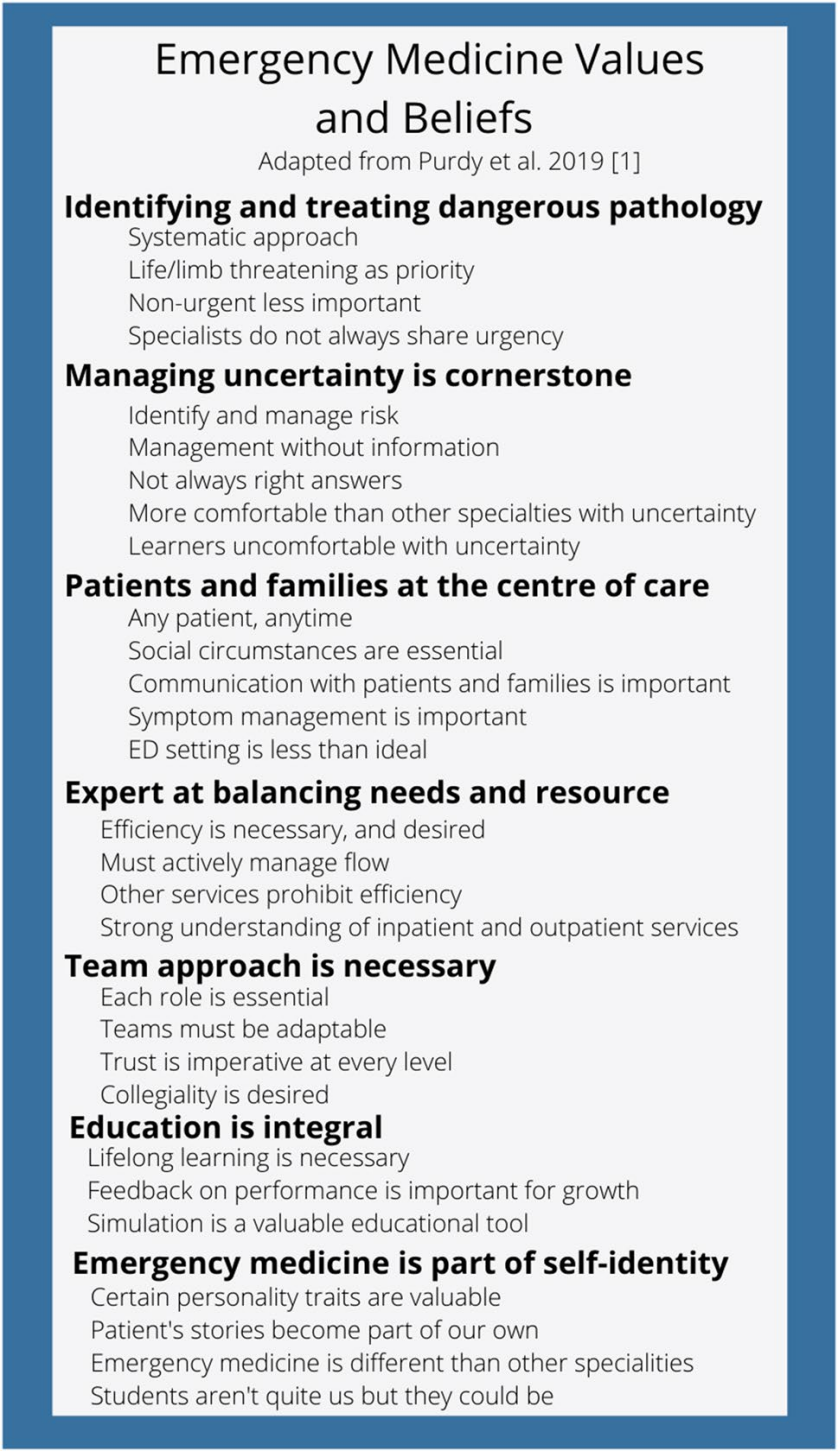
Emergency medicine value framework

Disasters efficiently magnify existing culture through several mechanisms (i.e., clear showcasing of value-based decisions, trade-offs, and priorities; more deliberate and open reflection on what is important; and a regression to intuitive behaviors) [9]. As such, the COVID-19 pandemic presented a unique opportunity to explore the values of EM in Canada. Increasingly, anthropologists, experts in the study of culture, are called upon to study hospital behavior to inform healthcare strategy [10–13]. Recently, they have sought to better understand the culture of EM and Purdy et al. has developed a framework that highlights seven core values as foundational to the beliefs and practices of the speciality (Fig. 1) [1–5]. This framework, serves as a tangible starting point for reflection around the EM community’s professional cultural experiences and challenges as magnified in the early phases of the COVID-19 pandemic.

Using data from an ethnography that informed the early departmental response to COVID-19, we sought to understand the impact of the pandemic on the manifestation of ED values in a Canadian tertiary care emergency department.

## Methods

We conducted a collaborative ethnography during the first 12 weeks of the COVID-19 pandemic. Collaborative ethnography is a method that partners directly with communities during design, data gathering, and analysis to study local culture in a way that is useful for those groups [14]. Our pragmatic approach was designed to provide local ED leadership with a weekly “pulse” from the floor to support real-time decision-making, while also being robust enough to provide in-depth insight into EM culture.

### Setting

Two sites associated with the Kingston Health Sciences Centre—Kingston General Hospital (KGH) ED and Hotel Dieu Hospital (HDH) Urgent Care were included. Kingston, ON, Canada has a population of approximately 136,000 and visits to both sites combined average ~ 100,000 per year. The study took place from March 15, 2020 to May 31, 2020.

### Creating the research team

A critical phase of collaborative ethnographies is engaging a local community in the design and conduct of the study. After consultation with department leadership by EP (EM resident and MSc in Applied Anthropology), the project was advertised. Any individuals in the department wishing to participate were included in the core research team. They assisted in design and had primary roles as data collectors in the collaborative ethnography process. Then, they were partners in the interpretation and distribution of results. The local individuals and roles at the time were EP (resident physician), SD (ED physician and quality improvement lead), LM (Nurse Educator), MP (Registered Nurse), KW (Registered Nurse and charge nurse), LR (ED physician and wellness lead), and DD (emergency physician and competency-based medical education lead). The external team members included DH (PhD Applied Anthropologist, specializing in disaster anthropology), RB (retired ED physician), GF (medical student), and HM (medical student).

### Participants

All staff (~ 300) working in the ED were included in participant observation and eligible for interviews. This included nurses, physicians, residents, unit clerks, respiratory therapists, environmental services experts, porters, administrators, and hospital leaders. Purposive sampling [15] of this group and open self-referral was used to identify participants for informal and formal interviews across the study period.

### Data collection

Field notes: All local research team members (EP, LM, MP, KW, LR, DD, and SD) recorded field notes during a convenience sampling of their shifts and from informal interviews using a template (Supplementary file 1). Observations occurred while on shift to minimize COVID exposures.

Interviews: EP conducted semi-structured interviews with participants (Supplementary file 2). The interviews were conducted by telephone, audio recorded, and transcribed. Participants had the opportunity to check transcripts.

Document review: Official documents including department daily/weekly updates, and clinical updates, and reports provided to ED leadership (Supplementary file 3) were archived and reviewed.

### Data analysis

Data were analyzed using theoretical thematic analysis in Nvivo 12.0 [16, 17]. Data were coded deductively by both EP and GF using the framework for EM culture (Fig. 1) weekly [1]. During the coding process they met regularly to discuss key findings, interactions, and additional reflections on the data and their positioning. These results were shared with the local study team and further interpretation discussed. The real-time analysis informed reports to ED leadership (Supplementary file 2) and shaped further data gathering.

### Member checking

At the end of the study period, collated results were summarized in a report and shared with the entire ED staff and leadership via email and placement around the department (Supplementary file 4). Informal verbal feedback was sought and two online town halls were hosted to solicit community perspective.

### Ethics

Queen’s University Health Sciences and Affiliated Teaching Hospital Research Ethics Board approval #6029355.

## Results

We conducted over 300 hrs of participant observation and informal interviews which informed over 47,000 words of field notes. EP conducted 42 semi-structured interviews including 9 attending physicians, 11 residents, 12 nurses, 6 core leadership team members, 1 paramedic, 2 porters, and 1 environmental services expert. Interviews ranged from 20 to 50 min. 57 departmental documents were archived and reviewed.

We readily identified aspects related to each of the values and beliefs from the EM values framework (Table 1). “The team approach” (Value 4) seemed protective and was even strengthened during the acute phase (Table 1). “Managing uncertainty” (Value 2) supported EM teams’ ability to navigate this tumultuous time. Most striking, however, were the threats to each of the other core values (Table 1) and the magnified tension between values. Below we outline how a system-wide response in the early weeks of COVID-19 exposed a problematic status quo, how unavoidable tensions become unmanageable, and how threats to values impact self-identity. During member checking, results resonated with colleagues and no significant changes were made to the interpretation of findings.

**Table 1:** Impact of COVID-19 on Values

Value		
Impact of COVID (alignment/threat)	Example	Evidence
*Value 1: Identifying and treating dangerous pathology is a key role of emergency medicine*		
COVID-19 disrupted usual approaches across all domains (environment, cognitive, procedural) which impacted the ability to identify and treat life-threatening conditions	Major changes were made to the systematic approach to treating dangerous pathology. Staff felt that the changes made to the management of all sick patients (i.e., resuscitation room layout, protocols, approach to respiratory failure) impacted their ability to do the basics of their job well	“…if I made you tie your shoes a different way and told you to do it, you're not gonna be able to do it…to me it's just that little switch in motor skill is very difficult, you know, not that it's a totally different motor skill, I think it's more cognitive.” – Attending (int 35)
	In caring for all patients, the risk of COVID-19 became front of mind but many expressed feelings of concern that this fundamentally changes the job	“..you can't just do what you usually did…that is everything from low-risk chest pain through to a broken arm that needs the sedation through to really bad respiratory failure…you can't just do the things that you got real comfortable doing and then enjoy the features of the job that you are really good at” – Attending (int 2)
	Environmental changes such as repurposing a section previously used for admitted patients was cognitively challenging for staff	"I couldn't wrap my head around sending sick cardiac patients back to section C, which used to be the space where we would be putting chronic care and crisis placements" – Nurse (int 24)
Personal safety precautions conflicted with providing timely emergent care	The priority of staff physical safety conflicted starkly with the belief that treating dangerous pathology is a priority. PPE impacted communication and slowed procedures – particularly with rapidly deteriorating patients which staff found this distressing	"Really the best care for this patient would have just been to do it [go into the room], but now we're protecting ourselves and subsequently patients, but I find that distressing in itself… it just took so long, it didn't feel good." – Resident (int 7)
Response to the acute threat of COVID-19 is more in keeping with EM strengths and energy than the ongoing management of the chronic realities of the situation	As the pandemic progressed it became clear that it was going to become a chronic problem, rather than an acute situation. With that realization energy faded. The staff’s relationship with the chronic realities of COVID-19 directly conflicted with preference for rapid problem-solving and disposition, and action in crisis	"We’ve entered a time now that I’ve heard described as “Chronic COVID” … and it feels to me, at least, like being stuck in a rut. "—Daily update May 4
		“We are great in a crisis—which was at the beginning of this mess. The more difficult part is now, when the crisis mode is over but normal isn’t coming back.” – Field Notes, direct quote from a resident
*Value 2: Managing uncertainty is a cornerstone of emergency medicine*		
Emergency culture is rooted in baseline comfort with uncertainty that was protective in early phases of the pandemic	Early on there was unprecedented uncertainty related to the disease, safety protocols, personal safety, and our collective approach. Members of the department seemed to pride themselves in being capable of navigating this uncertainty	“We are emergency doctors and nurses and team and so, sort of accepting and dealing with uncertainty is our specialty and we've been training for some years to do that.” – Resident (int 16)
Team strategies commonly used to manage uncertainty in individual cases were enacted at the department level to navigate periods of uncertainty as a community	Strategies that ED teams have used to manage uncertainty at the micro level for critically ill patients—i.e., pre-briefings, shared mental models—were adapted at a departmental level in the form of daily shift huddles and daily updates to manage this macro uncertainty. These strategies were particularly important during times when protocols were rapidly changing and were less common as uncertainty decreased	“It's nice that we're doing those little huddles, because I go back to work tomorrow and I've been off for 5 days, so I have no idea what's happened. Without that little huddle you kind of walk in there and you're a bit blind as to what's going on.” – Nurse (int 20)
Uncertainty related to personal safety was distressing, particularly with conflicting information sources	The intersection with personal safety that is not usually so central to work. This was a “hot focus” and source of distress for some and at least brain space for all others. Multiple sources of conflicting information (unions, media, leadership) were challenging and often stressful for staff to navigate	“I had so much doubt in our system and I have my union telling me one thing for proper protection and I have the hospital and Infection Control telling me another.” – Porter (int 29)
		“I'm worried that I don't actually know what the right answer is [about PPE and decontamination] …but my biggest fear is that I'm somehow going to get sick, infect my family and kill everyone in nursing homes.” – Attending (int 1)
*Value 3: Patients and Families are at the Center of Care*		
Strict ED visitor policies directly threatened the ability to provide patient-centered care	Staff usually rely on caregivers to provide collateral history or key observations regarding the patient’s status. Having this source of information removed was not only a hindrance to patient-centered care but also made it challenging to provide effective and efficient medical care. Many staff found this upsetting and they sometimes directly broke this policy when deemed necessary or appropriate and faced consequences doing so	"I feel like it's just so against everything that we've always done and really believe in. Like, it's just putting yourself in their position and not being able to see a loved one in such a time of need" – Nurse (int 3)
		“And if I think someone should come in then I will not back down…. I have been reported and I said, what the hell, you know? All you can do is, you can fire me.”- Charge Nurse (int 6)
COVID-19 prompted policies/protocols conflict with the value of patient-centered care	Changes to protocols, and associated inefficiencies, impacted the ability to provide high-quality and patient-centered care. Low-level friction costs across the system impeded the ability to provide streamlined care	“A very low risk for Covid patient met criteria for swabbing, but had a hip fracture…X-ray couldn't take her out of section C* (COVID area) but also couldn't do all the of the views without exposing everyone to significant radiation doses…so I could only get an AP pelvis, which showed a femoral neck fracture…but Ortho felt like they couldn't see her until she had full views and she couldn't get full views until she has a negative Covid swab… 8 h after I had diagnosed her hip fracture she was able to get the rest of her films and assessment by Orthopedics and admission…” – Attending (int 8)
Overcrowding is a direct threat to patients and staff	Early in the pandemic a dramatic reduction in patient volumes meant that ED boarding, which had been largely accepted as standard in the ED before COVID-19, rapidly resolved as resources were mobilized to move patients out of the department. This gave staff more time with patients and more appropriate ratios for care. Staff universally found this to be a positive for patients and providers alike	"Because you don't have the same time pressure to see patients…in a lot of ways it has kind of let me be a bit of a better doctor." – Attending (int 5)
		“It almost reminds me of the way it was maybe, you know, 30 years ago, 20 years ago in the Emergency Department where a patient was admitted and then went upstairs to their bed. There was none of this days and weeks in the Emergency” – Nurse (int 23)
	As volumes drifted back towards normal and ED boarding returned, desperate concerns about this practice for the safety and experience of patients came fast and loud from staff at all levels	“There's this concern that we are going to a week from now be back to having patients languishing in the emergency department with some associated concerns in that we don't actually have the space anymore to put those patients in hallways.” – EP Field notes
*Value 4: Emergency practitioners are expert at balancing needs and resources at a system level*		
Subtle changes in emergency department function from COVID-19 have significant impact on efficiency and flow	The first and one of the most important steps in balancing immediate needs and resources and coordinating flow is initial triage process – which was modified to meet infection control needs. Altering this process to a safe and workable solution required multiple iterations and immense flexibility from the nursing group	“They've changed to have the red and green triage nurse, so you're not just sending people back to section C*.. but before that you were blind because you'd call and they were full, but you would have no idea because you can't see the track.” – Nurse (int 19)
	Once in the department flow was further impacted by COVID-19. It became clear that even subtle changes in environment or procedures have substantial impact on the situational awareness and efficiency mindset needed to master this nebulous expert task	“Even though volumes are lower, I feel like efficiencies are really low at the moment so even though volumes are lower it's still hard to get through to patients in a reasonable time frame…” – Attending (int 14)
		“You always look at section A, B and D^*^ and you don't look at them independently because if you try and look at the entire department as one big EDIS^+^ screen it's just overwhelming and you don't, aren't able to make a plan whereas now I find myself pulling up C^*^ as much as any other department, if not more.” – Attending (int 14)
EM providers had to rapidly adapt to a changing healthcare landscape to preserve ability to link patients to resources they need	There were changes to how the emergency department could access outpatient services for patients. Details about changes to outpatient services available (i.e., dentistry, medical offices, shelters, homecare, etc.) were frequently included in the daily updates but were challenging to keep track of in practice. The broad impact of COVID-19 across the healthcare sector often came to a head in the emergency department and highlighted the department’s overwhelming role in helping patients navigate a complex system	“It [information about dentists]is in one of those emails but by the time it's next month you're gonna have to go through like, 60 emails to try and find the answer you have.” – Resident (int 27)
*Value 5: A team approach is necessary to providing high-quality care*		
Covid magnified that each team role is essential to the provision of safe care	As personal safety was threatened, the role of each team member and interconnectedness of those roles was on show which helped to emphasize the integral role of all team members in providing safe and efficient care. The threat of COVID further highlighted the need for psychological safety—and barriers to it—within the ED as individuals navigated advocating for the needs of patients, colleagues, and themselves	“It's not just, like the nurses do their things and the doctors do their things and the RTs [respiratory therapists] do their things, like everybody is super involved with everybody and like, definitely, like team player and take everyone's like, thoughts and stuff like that into consideration…”- Nurse (int 20)
		“I'm still not very happy about how some people [doctors and nurses] take on and put on and off their PPE, I know the docs as they're coming out of the trauma rooms, they're instructed on how to take things off, but I'm still seeing gloves on and the gown pulled over their head, that always drove me crazy…”–Environmental Services (int 22)
Teams need to be adaptable	As changes occurred daily, it became clear that operations would need to adapt rapidly. This was highlighted as a core value at both the individual and team levels. Frequent communication and interprofessional huddles facilitated team flexibility and challenged established hierarchies	“…what we were doing yesterday might not be applicable the next day so, you know, like having those little pre-briefings before shift change was able to bring everybody up to speed on kind of what happened that day, kind of refine some of the processes…” – Nurse (int 24)
Trust between groups and individuals is essential and is manifested and built through relationships	Trust was imperative to team function. Conflicting information and differing practices, particularly when personal safety was involved, threatened trust between groups. Trust seemed to be rooted in relationships. The charge nurses were identified as a node of trust in the department—with porters, nurses, environmental services, and residents all turning to them for guidance even when formal organizational ties were not direct	"…we trust what our docs say and that provides us with a lot of guidance but then when different attendings are providing different guidance, it just added some more confusion to the mix.” – Nurse (int 17)
		“People trust people they know versus the random email from incident command…you don’t even know who’s on that in Kingston…” – Resident (int 27)
Collegiality in day today practice and conflict management is prioritized	Emergency teams must be comfortable in stressful situations but conflict between colleagues is unavoidable. Prioritizing collegiality in management of this conflict seemed a value for many sometimes at the expense of frank dialog	“We're all on the same team and that it's not for one person to disprove the other and show them that one person is more right than the other, I think being able to listen to one another and.. not get too judgmental and not get too frustrated with one another because again, we're on the same team and we're all just trying to figure it out…” – Nurse (int 24)
*Value 6: Education is Integral to Emergency Medicine*		
Education/training resources and energy can be channeled in moments of need but only for a limited period of time	The novel threat of COVID-19 served as a strong motivator for the department to urgently address continuing education for attending physicians and teams. The prompted mandatory simulation-based airway training and in situ simulation that were unprecedented in scope but short-lived	"…Some of the staff have not intubated anybody in years and all of the sudden they're now having to do this high-stakes intubation with everybody looking at them through the glass and told to do it a different way than they normally would.” –Attending (int 12)
		“I think the sims, and really what has been a team thing supported by the department and the urgency of the situation has kind of contributed to buy-in, but I think people have valued that and that may be something that we can channel and continue on in the future.” – Attending (int 13)
The changes to educational activities have intended and unintended consequences	The intended purpose of simulation was to enhance team performance in real life situations, which many felt did occur, however there was also palpable tension between health care provider groups. The structure of simulations prioritized physician tasks which may have sent unintended messages to nursing and respiratory therapy staff that their role was somehow less important	"I do feel that they've been quite heavy on the physician role … I feel like the focus is always on the airway and always on getting from kind of the non-intubated to the intubated patient and I think that it would be nice to have the opportunity to also kind of explore more, kind of focus on the nursing like, how are the nurses gonna kinda facilitate the safest way of doing things" – Resident (int 21)
	The profound changes to standard educational programming (i.e., grand rounds, core rounds) allowed members of the community to reflect on the unintended outcomes of education as delivered before COVID. The inability to gather casually had significant drawbacks for the community	“The culture of our program I think evolves around us all sitting around in Richardson Lab Amphitheatre on Thursdays and you know, everybody kind of getting together, having their coffee, you know, having a nice presentation and I think it sort of brings people together so that's, that's gonna be a culture change if we are unable to do that in the future, or for a long time.”—EM Attending Field Notes
The call for clinicians to adapt practice and educate and/or maintain research productivity during crisis is a significant challenge	The threat of COVID-19 and associated public health realities resulted in educational adaptations including a new video conference-based core rounds and grand rounds. Informal mentorship was also negatively impacted. Many researchers in the department found it challenging to keep up productivity	“COVID has taxed teaching, not by making the environment difficult to be a good teacher in, I don't think, but instead by putting additional difficulties on cognitive load and personal reserve, which allow for enthusiasm, focus and dedication to being an educator.” – Attending (int 12)
		“I do worry about the resident experience throughout all this. I don't think, it doesn't keep me up at night, like losing, being absent from my children as a father does, but I think that I do worry about that this is a major threat to the educational experience.” – Attending (int 2)
*Value 7: Emergency Medicine is Part of Self Identity*		
COVID-19 resulted in the manifestation of traits and feelings that both support and conflict with self-identity	Some valued personality traits of those working in the ED—like adaptability, calmness, friendliness, and adaptability—were protective in the face of COVID-19	“I kind of try and keep as calm as possible and keep other people calm too you know, because I guess that's just my personality.” – Environmental services (int 22)
		“I think we're pretty adaptable so I think the trainees are pretty adaptable so I think that it will all be okay.” – Attending (int 2)
	Some, however, in the circumstances found themselves struggling with feelings that were not in keeping to their usual demeanor which seemed to be a potential threat to identity	"I know it's been difficult for everyone but then there's been a….little bit of negativity stemming from that and I think that it has to do with…I've sort of been feeling these things as well like, confusion and anxiety and um, and I'm not an anxious person.” – Nurse (int 34)
		“You can't just do the things that you got real comfortable doing and then enjoy the features of the job that you really love because they are different now, you know, they are just different. You can't even enjoy the interpersonal interactions.” – Attending (int 2)
	Throughout the study period Kingston did not have an influx of COVID-19 patients which posed another type of conflict in identity—some in our community felt underutilized and over celebrated	“This might be the defining medical event of our generation and it's kind of sad being an Emergency doctor, like part of me like really wants to be on the frontline or, you know, making a difference instead of actually doing less than I was doing before.”—Resident (int 34)
The physical threat associated with COVID-19 highlighted overlap between aspects of identity (i.e., home and work)	The intersection of risks of the job with family life and personal identity was sharp during the acute phase of the pandemic with many staff undergoing extensive decontamination protocols and arranging for emergency housing and contingency plans should they become infected	“Everything that I have with me that needs to go home is Virox cleaned before it goes into a clean part of my bag… anything that I'm wearing goes into a separate bag…” – Nurse (int 3)
		“I have a newborn at home so, number one I mean I'm worried about bringing, you know the virus home to me, like home to my family and home to my newborn. I mean I do worry about my family first and foremost, because they're all worried about me,” – Nurse (int 24)

*The emergency department has four sections A, B, C, and D: Section A is the resuscitation section, B is fast-track, section C became the COVID/influenza-like illness section, and D is ambulatory care. Before the pandemic hit, section C was largely used to board stable patients or patients awaiting long-term care

+ EDIS is the electronic medical record used in the emergency department

### The honeymoon period exposed a broken system

During the first few weeks of the COVID-19 crisis, the emergency department benefited significantly from system re-organization and increased resource allocation alleviating usual threats to ED values (i.e., boarding, overcrowding, lack of resources). This contrast allowed participants to recognize and describe many longstanding distressing daily realities that predated the pandemic. For example, they were troubled by the normalized practice of hallway medicine. As one nurse said,

“You should have a room for everybody. The hallway thing, I've seen it develop over the years and it's distressing because it's not a good thing and I know we're not the only place that does it, that's for sure, but how did we get to that?” – Nurse (int 23)

In the early phase of the crisis, stretchers in hallways were notably missing as system-wide resources relieved pressure. A glimpse of a system that was better functioning made going back to the disheartening realities of the usual system’s shortcomings more troublesome once misbalance returned.

“I have lived…in a medical system that has normalized emergency care for patients in what now, I'm actually realizing is a very problematic way [hallway medicine].”- EP Field notes

As time went on these gains, and the constructive system orientation towards the situation, disappeared. Not long after our study started, the brief COVID-19 honeymoon period ended. When that happened, emergency staff were left with significant challenges in negotiating a rapidly escalating and increasingly impossible balance of needs and resources to care for patients—a situation that simultaneously threatened multiple core values. The appetite to creatively optimize clinical environments and processes faded, further magnifying the misbalance for clinicians on the ground.

“[there is now] some strain around where to put patients now that volume has increased. There is limited acute/resuscitation hallway space, no offload, and now the subacute zones have the “long stay” patients back...” – LR Field notes

### Unavoidable tensions become unmanageable

Participants identified classic tensions between core values that are central to the practice of EM. For example, “*patients and families at the center of care*” (Value 3) often conflicts with the ability to “*balance needs and resources at a system level*”(Value 4). Participants suggested that the line differentiating expected tensions between these values and unacceptable stress from unmanageable conflicts between values was precarious and often crossed even before the pandemic. COVID-19 increased the likelihood of predictable tensions between values becoming demoralizingly unmanageable.

Visitor restrictions were one example. In the early days staff were bothered by, but could make sense of, the need for restrictions. The need to protect the system from uncertainty and disease was acceptable, despite potential harm to individual patients. But as time went on the threat to the value of patient and family centered care became more problematic. Distress ultimately came when providers felt that the potential benefit to the system no longer seemed to justify the individual harm.

“ …It’s just so against everything that we’ve always done and really believe in. …just putting yourself in their position and not being able to see a loved one in such a time of need.” – Nurse (int 3)

The requirement to put the system above individual patients, and the distress that caused staff, mirrored many of the more usual problematic challenges EM providers face when attempting to access care for patients in a system with limited resources—such as wanting to schedule an outpatient follow-up for a patient (to satisfy values 1, 2 and 3) but not having access to the appropriate clinic (Value 4).

### The threat to identity

Emergency providers value being good at their job and experience a profound heaviness, sometimes even manifesting as tears in our interviews, when that identity was threatened. This was most obvious when providers felt the work they were doing did not, or could not, align with their core values.

For example, though emergency providers pride themselves in “*managing uncertainty*” (Value 2), they were pushed to the limit as ever evolving infection control protocols required re-organization of resuscitation rooms, a change in triage protocols, and department layout. Staff found these environmental and process changes a direct obstacle to effectively “*identifying and treating dangerous pathology*”(Value 1).

“…everything got changed … even though I've been in there for weeks on end, I still don't know where stuff is…I think it also has affected some of the cases that I've been involved in.” – Attending (int 15)

On a more abstract level, underlying tension exists between “identifying and treating dangerous pathology” (Value 1), “managing uncertainty” (Value 2) and “EM is part of self-identity” (Value 7). COVID-19 highlighted how interconnected these values are. The threat of a new disease overtly challenged the traditional cognitive schema and systems that allow the care team to manage the uncertainty around critical illness effectively and this in turn impacted the confidence and self-worth of participants. The constant vigilance required to simultaneously manage uncertainty and expertly make critical diagnoses is not new in the COVID era but the increasing challenge in doing so emphasized how closely tied these capabilities are to self-identity and self-worth.

"You know, the two obvious things [that I worry about] are that I'm going to over-Covidize everything [think every presentation is COVID related]and just downplay something else and someone is going to have a bad outcome as a result of that..." – Attending (int 2)

## Discussion

### Interpretation of findings

The acute phase of the COVID-19 crisis offered a powerful lens for understanding the values and beliefs that underpin EM culture. We uncovered critical threats to EM values then saw the negative impact of holding a certain set of values but being unable to practice them. The readily identified threats to and tensions between core values can be used to contextualize current challenges facing the broader EM community. The results of this ethnography offer a helpful voice in merging the conversations related to EM systems and the rising levels of burnout.

### Comparison to previous studies

A recent Canadian Journal of Emergency Medicine editorial by Atkinson et al. outlined systems-based challenges threatening the current paradigm of EM including disproportionately high usage, lack of access to primary care, access block, and lack of long-term care funding [18]. The searing commentary resonated with emergency physicians across the country, as evidenced by robust online discussion and the second highest Altmetric Attention Score of all time for any CJEM article [19]. Meanwhile news outlets and researchers have identified alarming levels of discontent manifesting as burnout, exodus, and depression in up to 60% of the Canadian EM community—with obvious and dramatic implications for the national workforce [20–24]. The ethnography we present acts as a direct link between these systems frustrations and a looming workforce catastrophe.

The opportunity to have values and work align is a concept known as “value congruence”. It is essential to an effective and sustainable workforce. Across industries it is critical to longevity and operational success [6–9]. Unfortunately, our analysis spotlights a troubling degree of value incongruence in the current practice of EM that has been further magnified by COVID-19. Our study provides empirical data that supports the desperate need for realignment of the EM community’s values with actual work in EDs.

### Strengths and limitations

This was a single-site study in the early phases of the pandemic response in a geographic area with low COVID-19 case numbers at the time of the study. While many of our findings will be universal, some issues identified will be unique to our specific context. Some degree of turmoil may be explained by the unique stresses of COVID-19, which other studies have also identified [20, 26]. Undoubtedly, as the pandemic has evolved, the nature of the threat to values has changed. From collective experience, we speculate that that our findings related to value incongruences for our EM community are even more stark now than the acute phase that was the focus of this report.

We focused on areas of tension between and the threats to values in the ED but recognize that some aspects of EM work, mostly as they relate to teams, remain well aligned and even strengthened. We witnessed committed groups of people doing excellent work despite the profound challenges faced. These teams currently act as a buffer to the negative realities of value incongruence. Such team resilience is a phenomenon that should be further studied and organizationally harnessed.

### Clinical implications

Unfortunately, there are no simple fixes. We cannot offer a clear table of actions or easy to implement suggestions based on our findings. Issues that are as seemingly inconsequential as a poorly timed electronic medical record downtime to as significant as hospital funding models will impact whether EM values and practice align for clinicians on the ground. We do, however, hope that raising awareness about the concept of value congruence and providing the pragmatic structure of the EM values framework will be useful in several ways. *For individuals* in the face of ongoing threats, it can be a tool to cognitively frame experiences—admittedly a strategy that has limited impact and is only helpful in the short term. *For leaders*, it may provide practical scaffolding for unwavering defence of EM values for decisions at every management level. *For hospitals and systems*, it should provide urgency and direction for aligning funding, processes, and procedures with core values of those on the ground. Each of these requires a recognition of and conversation about the values and beliefs we hold, the importance of reconciling tensions that exist, and a brighter vision for our future. The alternate reality must also be faced. Under sustained threat, clinicians are likely to reconcile the incongruence experienced by shifting their values. If the system cannot match the current values of emergency providers, providers will match their values to the system. The culture of EM—the values, beliefs and practices that make us who we are—will change.

### Research implications

**Fig. 2:**
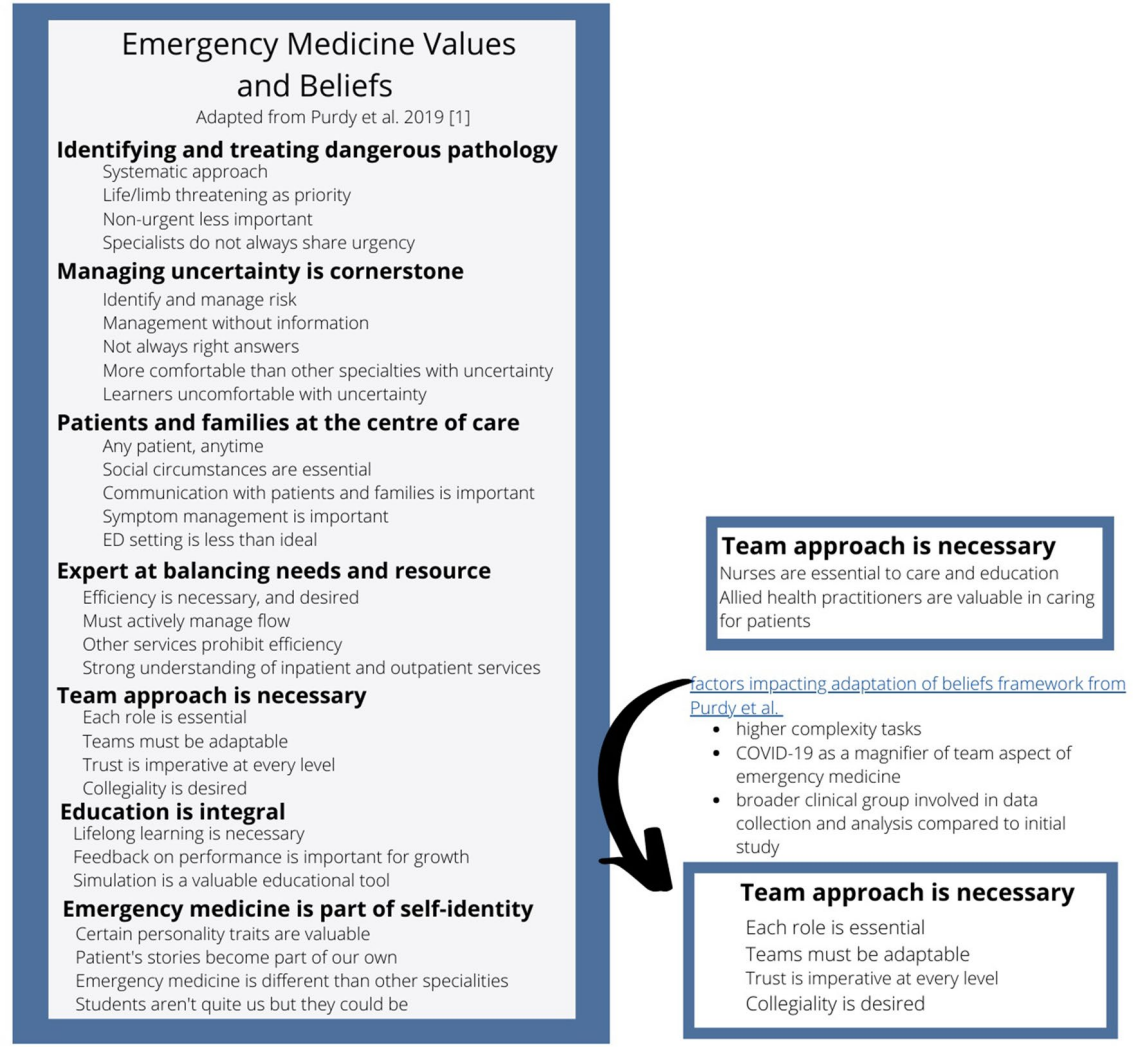
Refined emergency medicine value framework

Our single-site study should serve as a starting point for research related to value congruence, staff retention, and identifying health system policies that support alignment of work as experienced with the embodied core values of EM staff. Methodologically, we are the first group to apply the framework for EM culture (Fig. 1) by Purdy et al. in a clinical and Canadian context [1]. We did identify the need for changes to the framework as it related to teams (Fig. 2). This evolution highlights how understanding of culture is fluid and everchanging. There are likely to be local variations when applied in other sites. The framework is not meant to be a fundamental truth, rather a useful tool for exploring the culture of EM.

### Conclusion

COVID-19 has highlighted and compounded existing tensions and threats to the core values of EM. Realignment of the realities of ED work with staff values is needed to sustain the culture of EM in Canada.

## Supplementary Information

Below is the link to the electronic supplementary material.

Supplementary file1 (PDF 111 KB)

Supplementary file2 (PDF 163 KB)

Supplementary file3 (PDF 85 KB)

Supplementary file4 (PDF 6210 KB)
