# Supplementary material for: COVID-19 has heightened tensions between and exposed threats to core values of emergency medicine
Source: CJEM. 2022 Sep 10;24(6):585–98. doi: 10.1007/s43678-022-00383-0 (PMC9463050; doi:10.1007/s43678-022-00383-0)
Supplement: Supplementary file 1 — Supplementary file1 (PDF 111 KB) [file 43678_2022_383_MOESM1_ESM.pdf]

- Tell me about your experiences working in the emergency department during COVID-19
- Tell; me about something that is working well?
- What do you think we could do just 1% better?
- What have been your biggest concerns and challenges so far?
- Do you feel like you are being adequately protected and supported?
- How might the department support you best in your role?
- How do you balance doing your job, with protecting yourself, concern about your family?

+Snowball: others to contact for more info?

Cognitive

Social

Envt/Space/Flow

Linguistic

Other (communication/safety/wellness/change)
