# Supplementary material for: COVID-19 has heightened tensions between and exposed threats to core values of emergency medicine
Source: CJEM. 2022 Sep 10;24(6):585–98. doi: 10.1007/s43678-022-00383-0 (PMC9463050; doi:10.1007/s43678-022-00383-0)
Supplement: Supplementary file 2 — Supplementary file2 (PDF 163 KB) [file 43678_2022_383_MOESM2_ESM.pdf]

Working Together in Crisis – Weekly Report 2

Date: March 28, 2020 Delivered to:

Created by: Eve Purdy + WTIC Team (Brison, Caudle, Dagnone, Douglas, Hill, McDonough, Rang, Wood)

Social

Overall-*"Morale has never been so high"*-nurse

-Some more discontent this week – seems to be compounded by airway sims depending on how led, dynamic situations (hard to avoid), and fundamental things like access to PPE and scrubs.
-some (MDs/RNs) feeling overmanaged instead of supported around airway sims.
-Security feeling undervalued/under-protected re: access to PPE. Same situation with HDH nursing staff and access to scrubs.
-Residents – high morale w manageable work loads/public recognition
-nightly nursing Zoom meetings are nice, can re-evaulate frequency
-HDH sometimes feels "out of the loop" – a.m. huddles are particularly valued and appreciated by nursing staff here.

Cognitive/Process

-need finalized Queen's ED airway protocol so people feel agency and ability to practice) and complete shared mental model. "perfect the enemy of good"
-information overload, difficult to stay up to date. Also Challenging to keep other diagnoses in mind. Other dx being missed in patients going up to floor.
-concerns from RNs that sims catered towards MDs and not team (other issues that come up with COVID not just intubation – i.e. how do you get lab samples to lab without contaminating, transferring to ICU etc)
-MDs unsure of protocol for use of CPAP in ED and when this is appropriate.
-+++issues with transfer of intubated ?covid patients to ICU. When/how this happens.

Environmental

-Section C is a cognitively very challenging place for physicians. There are sick patients, but no assigned physician and not used to this being a place where sick patients can be. Limited equipment and geographically distant.
-Section C is a challenging place for nurses on lots of levels. Excellent nurses but minimal support and long time without interaction with others from the "outside"
-No glidescope at HDH yet
-++ clutter in section A1,2,3/ B hallway – this needs to be more organized
-unclear how to triage between section A/C.
-admin reports increased efficiency (ie. able to implement changes quickly)

Safety/Vulnerability

-ongoing significant concerns about PPE. Some sense that messages from the top are not in keeping with reality of situation. Conflicting messages about PPE recommendations – particularly from ONA increase that distrust.
-questions from nursing re: masks during breaks (what is safest practice while conserving PPE)
-many staff sourcing their own re-usable respirators
-RNs nervous due to differing swabbing criteria; pt in ED for hours unprotected then swabbed
-residents fearful, unsure of where to stay if get sick (reported barrier per Donald Gordon = cost)
-pregnant nurses feeling ++++ vulnerable

Good Ideas from the Ground

-Clear criteria for deciding whether patients go to section C/A

-HDH/unit clerk scrub access

- team/day huddle, followed by immediate sim rather than walk through

-drug packs, IV packs for COVID + rooms that are pre-made up

-Tables in A1/2/3 so that RNs have place for equipment

-multibedroom resident apartment for those on self-isolation/quarantine/COVID +ve

-NIV decision making (ie. Set of criteria for low/ med/ high COVID risk or 2x physician decision making)

- fun, COVID unrelated videos/ photos for destressing in break room

-PPE for security

-involve security/unit clerks/environmental services in huddles

-Scrubs for HDH nurses

-Sims of deteriorating section C patient/transfer to ICU/triage

Communication

-daily update email to MDs is totally on point. Nice balance of humour. Pictures appreciated and morale building. Appropriate information.
-nurses would appreciate info about #'s of +ve's in community
-overall communication is somewhat more tense (charge nurse to nursing, nurses to MDs) likely from increased anxiety.
-despite initial request for something other than clean vs dirty, that seems to have stuck and is the easiest to comprehend nomenclature

Top 5 Recommendations

1. **Huddle led by section A doc or Charge Nurse** (not others i.e. airway experts/administrators): focus on team specific morale/processes for the day. Team can then do an intubation sim (this should also be led by A doc with ED airway protocol – experts for support if needed).
2. Consider **radical/complete transparency** about projected access to **PPE** + ongoing efforts to secure. This could prove significantly problematic in terms of trust and commitment if not done and down the line and there is a shortage. THIS IS THE NUMBER ONE CONCERN OF STAFF.
3. Workshop **triage to section A/C, patient deterioration**, **transport to ICU** not just intubation. We can optimize how sick resp patients will be cared for safely throughout care and how they will be transported out of department as there were issues on all these fronts this week.
4. Prioritize **inclusion of non-MD/RN** roles in department morale: include security in huddle/advocate for their access to PPE, unit clerk scrub access (or purchase them scrubs if no hospital access), environmental services thank you/inclusion
5.Given the large degree of standardization we are doing around airway we need a **glidescope accessible at the hotel dieu** ASAP.
[illegible]

*Data curated from 12 structured shift reflections (7 sources – nursing + MD), 15+ informal interviews, observation from peri-departmental activities and communications
