# Supplementary material for: COVID-19 has heightened tensions between and exposed threats to core values of emergency medicine
Source: CJEM. 2022 Sep 10;24(6):585–98. doi: 10.1007/s43678-022-00383-0 (PMC9463050; doi:10.1007/s43678-022-00383-0)
Supplement: Supplementary file 3 — Supplementary file3 (PDF 85 KB) [file 43678_2022_383_MOESM3_ESM.pdf]

Semi-Structured Interview Guide

"I am going to ask you questions about your experience in the emergency department. This conversation will be audio recorded and transcribed. Once transcribed the audio recording will be deleted. Any identifying information will be removed. I expect that we will talk for 15-30 minutes. Do you have any questions? Are you happy to proceed?"

-Tell me about your experience working in the emergency department during COVID 19.
    -explore negatives
    -explore positives
-Describe your role in the department? Has that changed during the COVID 19 response? How?
-Describe has gone well in the department response? For you personally?
-What could go just 1% better?
-Tell me about your biggest ongoing worries?
