# Supplementary material for: COVID-19 has heightened tensions between and exposed threats to core values of emergency medicine
Source: CJEM. 2022 Sep 10;24(6):585–98. doi: 10.1007/s43678-022-00383-0 (PMC9463050; doi:10.1007/s43678-022-00383-0)
Supplement: Supplementary file 4 — Supplementary file4 (PDF 6210 KB) [file 43678_2022_383_MOESM4_ESM.pdf]

NOVEMBER 2020

COVID-19 ED Ethnography

Part 1: the pandemic as a moment of cultural magnification, creativity, and transformation

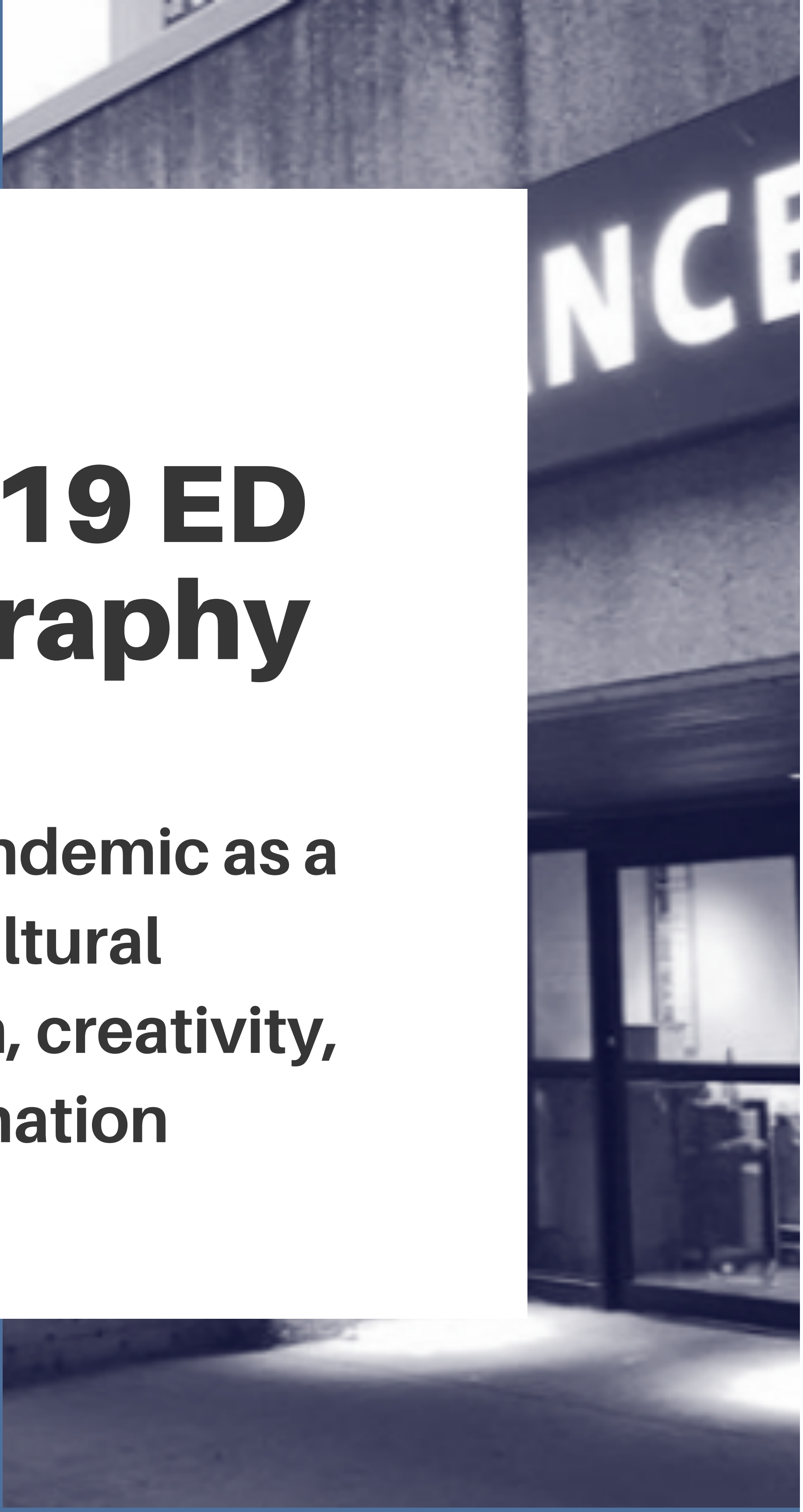

Prepared by Eve Purdy and Gillian Forster
For the ED COVID Ethnography Team

The Project

Dear ED Team,

Thank you to all who participated in the ED COVID-19 rapid response ethnography that took place in the spring. We conducted over 50 formal interviews, gathered hundreds of pages of field notes based on observation and informal interviews, and reviewed organizational documents during the acute phase of the pandemic response. During that time period we rapidly analyzed that data to provide weekly summaries and recommendations to the hardworking leadership team with the intent of supporting their ability to make real-time decisions. If you have any feedback on your involvement in this process we would be keen to hear your thoughts.

In addition to supporting department response, we planned analysis of this data to better understand how organizations respond to crisis and what such a dramatic moment in time can tell us about the culture of emergency medicine. Over a series of reports we will present these findings to you and be seeking your thoughts about whether our analysis resonates and to sort out what we have missed. Stay tuned for the announcement of virtual opportunities to tell us what you think or email us (epurdy@qmed.ca or stuart.douglas@queensu.ca) anytime!

This first in a series of reports is an analysis of ED values and beliefs during COVID.

Again, thank you for letting us into your experience and for the hard work you do every day, especially during these trying times. It was a privilege to curate your stories.

Eve Purdy, Stuart Douglas, Louise Rang, Krista Wood, Laura McDonough, Damon Dagnone, Meredith Powell, Doug Henry, Gillian Forster, Hayley Manlove, and Rob Brison

The planned outputs

- Weekly real-time reports to leadership during acute phase of COVID-19 (completed)
- **ED CULTURE: how does COVID-19 expose, challenge, and shape our collective values and beliefs? (this report)**
- RELATIONAL COORDINATION: how do shared knowledge, goals, mutual respect, and communication manifest during a response to a pandemic
- RAPID CYCLE ETHNOGRAPHY DURING CRISIS: reporting and reflection on the process

How do we study ED Culture?

...and why would we during a pandemic?

Culture is a rather nebulous term but really it boils down to the **values, beliefs, and practices** shared by a group of people. Usually the story of a culture (an ethnography) is created by gathering and analyzing many different types of data - interviews, observations, document review - over a period of time. In some moments, such as during educational activities...or....during a pandemic, the culture of a group can be magnified - making it easier to see. These moments may also shape culture in meaningful ways. The pandemic is what we could call a moment of cultural compression for our department. The perfect opportunity to understand who we are and think together about how COVID-19 might influence who we become.

Researchers, including EP, had previously built a framework for ED culture that identified a set of relevant values and beliefs. Our research team sought to 1) apply this model to a new ED group to test and further refine the framework and 2) understand how the pandemic might challenge, support, or impact ED culture in new ways.

The data gathered over 11 weeks (March 14- May 21) included:

43

formal interviews

nurses, physicians, residents, porters, admin, environmental services
leadership, paramedic

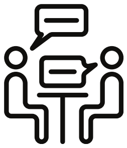

47,000

words

from field notes written by 7 members of the research team based on structured observation and informal interviews

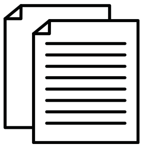

57

documents

updates, protocols, news stories, educational resources

Summary of findings

Each of the **values and many of the beliefs from the original framework were present** within our dataset. We will review each of these values in the context of our community further in this report. When coding for additional values we identified **significant deficit in the construct related to team approach** in the original framework. It was far too simple and we have proposed an alternative. Interestingly the pandemic both supported and directly challenged ED values. **Physical safety considerations impacted each value** in a way that may account for ongoing practice and identity challenges in the more chronic phases of COVID. Studying culture in a pandemic magnified how **values can conflict with each other** which warrants more exploration by those interested in promoting practice sustainability and staff wellness, and managers seeking to coordinate systems that reduce that internal conflict. We found that COVID-19 was a moment of cultural **MAGNIFICATION, CREATIVITY,** and provides a potential accelerant for **TRANSFORMATION.**

Original Framework

Identifying and treating dangerous pathology
- Systematic approach
- Life/limb threatening as priority
- Non-urgent less important
- Specialists do not always share urgency

Managing uncertainty is cornerstone
- Identify and manage risk
- Management without information
- Not always right answers
- More comfortable than other specialties with uncertainty
- Learners uncomfortable with uncertainty

Patients and families at the centre of care
- Any patient, anytime
- Social circumstances are essential
- Communication with patients and families is important
- Symptom management is important
- ED setting is less than ideal

Expert at balancing needs and resource
- Efficiency is necessary, and desired
- Must actively manage flow
- Other services prohibit efficiency
- Strong understanding of inpatient and outpatient services

Team approach is necessary
- Nurses are essential to care and education
- Allied health practitioners are valuable in caring for patients

Education is integral
- Lifelong learning is necessary
- Feedback on performance is important for growth
- Simulation is a valuable educational tool

Emergency medicine is part of self-identity
- Certain personality traits are valuable
- Patient's stories become part of our own
- Emergency medicine is different than other specialities
- Students aren't quite us but they could be

Modifications to the Framework

- maturation of the team approach value and beliefs to:

  Team approach is necessary
  - Each role is essential
  - Teams must be adaptable
  - Trust is imperative at every level
  - Collegiality is desired
- in a pandemic the framework clearly operates in a wider context of safety which can modify each value to varying degrees
- increased appreciation for the tension that can exist between these goals, especially in times of crisis

a moment of cultural...

magnification

The early stage of COVID-19 was a time when the values and beliefs were easier to identify than in normal times. We gained deeper understanding around how values manifest within our group through specific practices. We uncovered more about the values and beliefs related to teamwork than previously understood.

The pandemic provided a specific lens to explore tension between these values, especially during times of crisis. The complicating factor of physical safety impacted each value in important ways. We saw how the threat of COVID played to our group strengths but also challenged our core values in fundamental ways.

This moment of cultural magnification serves as a reflection point to think about who we are.

creativity

The pressures of the situation resulted in the necessity, ability, and energy for the group to re-imagine and modify previously entrenched practices. Early on, traditional barriers and hierarchies associated with change were minimized which resulted in a setting ripe for cultural creativity across domains (environmental, social, cognitive). Some of these changes have stayed, some were temporary.

Areas of exploration included modifications to triage, resuscitation, physical layout, departmental policies, access to outpatient services, flow in the department, uniforms, team huddles, patterns of relationships each other and with other services, educational events, and social events. In each of these operational and social experiments we got a glimpse of what our community felt was important, largely driven by an effort to preserve values and sometimes even with a vision of a future better aligned with those core values.

transformation?

As we settle into a new normal, we are left with a moment to ponder how this significant event and moment of associated cultural creativity might result in long lasting transformation for our group... Permanent disruption to protocols, environment, physical safety, relationships, identity, and realities associated with patient/family interactions will have long lasting impact on our core values. There will be ongoing tension between and support of those values. What new ideas and practices do we want to keep? How as individuals and a community can we align our practices with our values moving forward in this new world? What does our future look like? Who do we want to become?

Key questions as you read

While working your way through this report , consider....

- Do these values resonate with your experience working in the emergency department and/or during the pandemic specifically ?
- How do these values conflict with each other? What does that mean for our day to day practice? For our leadership? Our system? For you personally?
- Are there experiences or feelings during the response to COVID-19 that are not captured?
- What fits well with your experience?
- What have we missed? What isn't quite right?
- What surprises you?

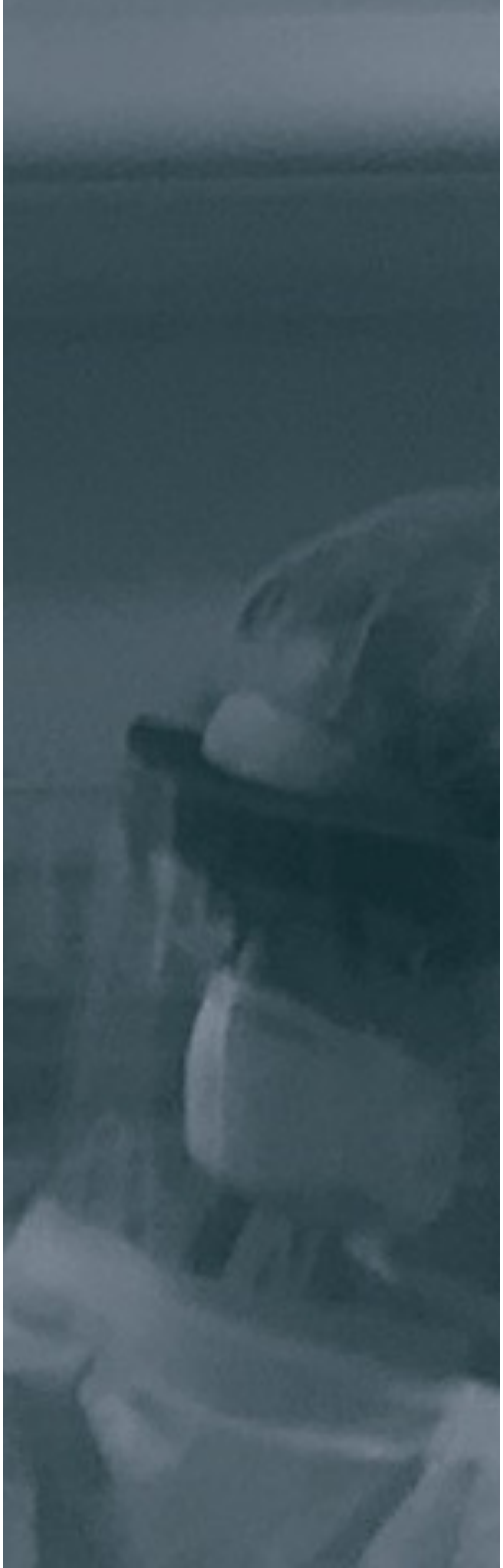

Identifying and treating dangerous pathology is a key role

During the pandemic, major changes were made to the systematic approach to treating dangerous pathology. The changes made to the management of all sick patients (i.e resus room layout, protocols, approach to resp failure) required an increased cognitive load which staff found tiring.

> *"...if I made you tie your shoes a different way and told you to do it, you're not gonna be able to do it...to me it's just* ***that little switch in motor skill is very difficult, you know, not that it's a totally different motor skill, I think it's more cognitive."*** – EM Attending

A core emergency medicine task, intubation and airway management, evolved rapidly from the clinicians' choice to an algorithmic and protocolized procedure in the face of this new disease process.

> *"A standardized approach helps us by* ***decreasing cognitive load*** *– fewer decisions frees brain cells to concentrate on technique. It creates a shared mental model so the entire team knows what to expect..."* - KHSC Covid Airway Management Protocol

COVID-19 became front of mind but many expressed feelings of concern that this may result in other life-threatening diseases being missed.

> *"You know, the two obvious things [that I worry about]are that I'm going to* ***over-Covidize everything*** *and just downplay something else and someone is going to have a bad outcome as a result of that..."* - EM Attending

The triaging of patients who screen COVID +ve but who are sick was challenging because section C is not where acutely unwell patients have historically been cared for making it cognitively and geographically difficult.

> *"I couldn't wrap my head around sending sick cardiac patients back to section C, which used to be you know, the space where we would be putting like, chronic care and crisis placements, right?"* - EM Nurse

Overall initially volumes were dramatically lower than baseline. HCPs had conflicting views as some were happy that only patients who were in "real emergencies" were showing up to the ED while others were concerned that those with dangerous pathology were staying home. There was a perceived increase in the amount of primary and follow up care provided as other offices were closed.

Physical safety conflicted largely with the belief that treating dangerous pathology is a priority. PPE impacted communication and slowed procedures.

> *"Really the best care for this patient would have just been to do [go into the room] it, but now* ***we're protecting ourselves*** *and subsequently patients, but I find that distressing in itself... but it just took so long, it didn't feel good."* -EM Resident

As the pandemic progressed it became clear that it was going to become a chronic problem, rather than an acute situation. With that realization enthusiasm faded. In the ED we usually work to determine what is acutely wrong with patients. Once stable, they are either sent home or admitted. Chronic COVID, it turns out, cannot be dispositioned from the ED - which directly conflicts with a value preference for rapid problem-solving and disposition, and action in crisis.

> *"We've entered a time now that I've heard described as "Chronic COVID"... and it feels to me, at least, like being stuck in a rut. "* - Daily update May 4

> ***"We are great in a crisis - which was at the beginning of this mess. The more difficult part is now, when the crisis mode is over but normal isn't coming back."*** **- EM resident**

Key Findings

- COVID-19 **disrupted usual approaches** across all domains (environment, cognitive, procedural)
- **Personal safety precautions** sometimes conflicted with providing timely emergent care
- Response to the **acute threat** of COVID-19 is more in keeping with emergency medicine strengths and energy than the ongoing management of the chronic realities of the situation.

Managing uncertainty is a cornerstone of EM

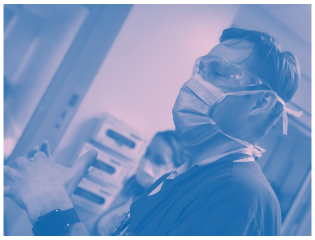

" We are emergency doctors and nurses and team and so, sort of ***accepting and dealing with uncertainty is our specialty*** *and we've been training for some years to do that."* - Resident

The emergency department response to COVID-19 highlighted that a cornerstone of our role is to manage uncertainty – this time however, it was not only obvious at the individual patient level, but on dramatic show at a systems level too. During the early stages of our ethnography there was unprecedented uncertainty related to the disease, safety protocols, personal safety, and our collective approach.

"Initially when everything first started ***I felt a lot of anxiety because every time I would come into my shift things would change****, what we're supposed to wear, what we're supposed to do, how we're supposed to screen people..."* - Nurse

We saw that common strategies that ED teams have used historically to manage uncertainty at the micro level for critically ill patients – i.e. pre-briefings, shared mental models – were adapted at a departmental level in the form of daily shift huddles and daily updates to manage this macro uncertainty. These strategies were particularly important during times when protocols were rapidly changing and were less common as uncertainty decreased.

"It's nice that ***we're doing those little huddles****, because I go back to work tomorrow and I've been off for 5 days, so I have no idea what's happened in the last 5 days, so without that little huddle you kind of walk in there and you're a bit blind as to what's going on."* - Nurse

Early in the pandemic there was the sense that the emergency department was helping consulting services manage uncertainty with numerous examples of the ED walking consulting services through new protocols or fear and uncertainty related to COVID-19.

"I think there is a fair amount of ***fear in other places*** *because a lot of you know, people are getting information from all sorts of mainstream and alternative medias that can give you a much more dire picture of what's going on..."* – EM Attending

A complicating feature of uncertainty associated with the pandemic was the intersection with personal safety that is not usually so central to work. Understandably this was a "hot focus" and source of distress for some and at least brain space for many. Multiple sources of conflicting information (unions, media, leadership) was challenging and often stressful for staff to navigate. As part of managing the risk associated with this intersection of uncertainty residents were not involved in the management of critically ill patients.

"I had so much doubt in our system and I have my ***union telling me one thing for proper protection and I have the hospital and Infection Control telling me another.****"* – Porter

"I'm worried that ***I don't actually know what the right answer is.*** *[about PPE and decontamination]...but my biggest fear is that I'm somehow going to get sick, infect my family and kill everyone in nursing homes."* – EM Attending

Key Findings

- Emergency culture is rooted in **baseline comfort with uncertainty** that is protective during a a pandemic
- Uncertainty related to **personal safety** can be distressing, particularly with conflicting information sources
- Team strategies commonly used to manage uncertainty in individual cases were enacted at the department level to **navigate periods of uncertainty as a community**

Patients and families are at the center of care

""Patients who are relying on their family to, you know, help prevent delirium, the heartbreaking cases of patients who are getting devastating diagnoses or dying, ***I think that it's really interesting to see the really humanistic smaller gears that make this whole machine move and now that those have been removed, the ways it falls apart.***" -Resident

Throughout the pandemic patients being at the center of care conflicted with maintaining the physical safety. While it was clear that patient care and symptom management continued to be a priority, they were often delayed by protocols such as awaiting negative COVID swabs or donning of PPE. One example of this was a patient who came in with a suspected hip fracture who, because of COVID-related concerns, was unable to receive the appropriate x-rays as per the radiology protocols that day. This resulted in the patient being unable to be seen by Ortho for over 8hrs. Instead of receiving patient-centered care which would have revolved around the diagnosis, and treatment of her hip fracture and analgesia with a femoral block, the patient's treatment was delayed while she awaited a negative COVID swab.

"People really wanna do it right and I think that's the main reason why it took a long time is because ***we forgot about the patient because we wanted to do this task*** *right."* – EM resident

Strict visitor polices conflict with the value that patients and families are at the centre of care. Many staff found this upsetting and they sometimes directly broke this policy when deemed necessary or appropriate and faced consequences doing so.

"I feel like ***it's just so against everything that we've always done and really believe i****n. Like, it's just putting yourself in their position and not being able to see a loved one in such a time of need"* - EM Nurse

"And ***if I think someone should come in then I will not back down....*** *I have been reported and I said, what the hell, you know? All you can do is, you can fire me."*- Charge Nurse

In contrast some nurses and physicians found caring for patients easier without patients' families present and this may be evidence of conflicting values between emergency medicine providers.

"In general, I find visitors might be helpful for the patient but is usually they're a bit of a barrier for us as physicians, so not having them there it is actually, I find, makes patient care a little bit easier." – EM attending

It is possible that by removing family members, some individuals found they could focus on other values such as balancing needs and resources by providing care more efficiently. Nevertheless, with the absence of family members, the ED is not an ideal environment for most patients.

Communication with patients was both challenged and promoted during the acute phase of the pandemic. Physical safety and the requirement of PPE inhibited communication in the form of body language and facial expressions.

"There's no question that there's a lot of nuance in body language and facial expressions from both the interviewer and interviewee that are not going to be picked up on ..." – EM attending

While some staff expressed that some aspects of communication improved.

"Because you don't have the same time pressure to see patients...in a lot of ways it has kind of let me be a bit of a better doctor." - EM Attending

For patients one benefit was the initial resolution of overcrowding. Those short term gains have largely been lost as volumes have returned to normal but inefficiencies have remained.

There's this concern that we are going to a week from now be back to having patients languishing in the emergency department with some associated concerns in that we don't actually have the space anymore to put those patients in hallways." - Physician fieldnotes

Key Findings

- Many **COVID prompted policies/protocols conflict with the value of patient-centered care** with varying reactions to this conflict by staff
- **Overcrowding**, initially relieved by a coordinated systemic response, **is a direct threat** to patients and staff
- The ED is **less patient-centred** than before COVID

Expert at balancing needs and resources at a system level

*"You always look at A, B and D and you don't look at them independently because if you try and look at the entire department as one big EDIS screen **it's just overwhelming and you don't, aren't able to make a plan** whereas now I find myself pulling up C as much as any other department, if not more."* – EM Attending

*"Unless I specifically call and ask for help, it's tough to get that sometimes back there [HDH COVID hallway]. **People forget about you** because you're out of the way."* – HDH Nurse

The emergency department often serves as a jumping off point for patients' care – the chain-link between patients and the services they ultimately need in the moment and down the road. This requires efficient practice within the department and extensive knowledge, resources, and facilitation of care within the system. COVID-19 has dramatic effects on managing flow at both levels. The early phases of the pandemic also provided conditions that hinted at what coordination with other services might look in a more ideal world.

The first and one of the most important steps in balancing immediate needs and resources is the tried and true triage process – which was modified to meet infection control needs. Altering this process to a safe and workable solution required multiple iterations and immense flexibility from the nursing group.

*"They've changed to have like, the red and green triage nurse, so you're not just like, sending people back to C, but before that, like, **you were blind** because you'd call and they, like were full, but you would have no idea because you can't see the track."* – EM Nurse

Once in the department the team further manages needs and resources by managing "flow" another essential concept impacted by the COVID-19. Flow requires a situational awareness and efficiency mindset that is actually challenging to encapsulate but COVID-19 made clear that even subtle changes in environment or procedure have substantial impact on this nebulous concept.

*"Even though volumes are lower, **I feel like efficiencies are really low** at the moment so even though volumes are lower it's still hard to get through to patients in a reasonable time frame..."* – EM Attending

Emergency medicine is at the interface with other groups that impact efficiency and the ability to balance needs and resources. Distinct successes (i.e. decreased red-tape for change, goodwill from consulting services) and challenges (i.e. more difficult outpatient access) at these interfaces were precipitated by the pandemic. Most glaringly, an early mobilization of resources at a system level to free up beds gave a glimpse of how an emergency department can function without hospital overcrowding. In this state, managing flow was a less active process.

Some gains in efficiency (i.e direct to psychiatry triage, reduction in overcrowding/boarding) were short-lived once the acute phase ended.

*"It almost reminds me of the way it was maybe, you know, 30 years ago, 20 years ago in the Emergency Department where a **patient was admitted and then went upstairs to their bed.** There was none of this days and weeks in the Emergency"* – EM Nurse

On a less noticeable level there were changes to how emergency physicians could access outpatient services for patients. Details about changes to outpatient services available (i.e. dentistry, medical offices, shelters, homecare, etc) were frequently included in the daily updates but were sometimes challenging to keep track of in practice.

"I'm like, it's [information about dentists] in one of those emails but by the time it's next month you're gonna have to go through like, 60 emails to try and find the answer you have." – EM Resident

The broad impact of COVID-19 across the healthcare sector often came to a head in the emergency department and highlighted the department's crucial role in helping patients navigate a complex system.

Key Findings

- **Subtle changes** in emergency department function **have significant impact** on perceived efficiency and flow
- EM providers had to **rapidly adapt to a changing healthcare landscape** to preserve ability to link patients to resources they need

A team approach is necessary to providing high quality care

Throughout the acute phases of the pandemic, EM teams were put under stress and this resulted in both increased bonding and trust within the team as a whole while simultaneously increasing the frequency of conflict between individuals. Some of this can be attributed to the uncertainty and lack of protocols leading to differing ideas as individuals gathered information from different sources. The situation occasionally highlighted and sometimes challenged existing hierarchies, sources of power, and fracture lines between groups. The strong pre-existing relationships within the ED played an integral role in allowing the team to approach this challenge as a unit.

"Emergency Department family is more actually in keeping with a family than it is just a group of colleagues...I think it just demonstrates that the organizations and departments that focus on the well-being and relationships of their colleagues will exceed when put into stressful circumstances" - EM Resident

Emergency teams must be comfortable in stressful situations but conflict between colleagues is unavoidable. Prioritizing collegiality in management of this conflict seemed a value for many occasionally at the expense of frank dialogue.

"We're all on the same team and that it's not for one person to disprove the other and show them that one person is more right than the other, I think, you know, being able to listen to one another and, and not, I don't know, not get too judgmental and not get too frustrated with one another because again, we're on the same team and we're all just trying to figure it out..." - EM Nurse

Key Findings

- **Each role** is essential to the provision of safe care
- Teams need to be **adaptable**
- **Trust** between groups and individuals is essential and is manifested and built through relationships
- **Collegiality** in day today practice and conflict management is prioritized

As personal safety was threatened, the role of each team member and interconnectedness of those roles was on show which helped to emphasize the integral role of all team members in providing safe and efficient care. The threat of COVID further highlighted the need for psychological safety - and barriers to it - within the ED as individuals navigated advocating for the needs of patients, colleagues, and themselves.

"I'm still not very happy about how some people [doctors and nurses] *take on and put on and off their PPE, I know the docs as they're coming out of the trauma rooms, they're instructed on how to take things off, but I'm still seeing gloves on and the gown pulled over their head, that always drove me crazy... You take your gloves off first, you gotta do your learning."* –Environmental Services

"It's not just, like the nurses do their things and the doctors do their things and the RTs do their things, like everybody is super involved with everybody and like, definitely, like team player and take everyone's like, thoughts and stuff like that into consideration..." – EM Nurse

As changes occurred daily, it became clear that operations would need to adapt rapidly. This was highlighted as a core value at both the individual and team levels. Frequent communication and interprofessional huddles facilitated team flexibility and challenged established hierarchies..

"...what we were doing yesterday might not be applicable the next day so, you know, like having those little pre-briefings before shift change was able to bring everybody up to speed on kind of what happened that day, kind of refine some of the processes..." – EM Nurse

Lastly, the pandemic highlighted that trust was imperative to team function. Conflicting information and differing practices, particularly when personal safety was involved, threatened trust between groups. Trust seemed to be rooted in relationships. The charge nurses were identified as a node of trust in the department – with porters, nurses, environmental services, and residents all turning to them for guidance even when formal organizational ties were not direct.

"...we trust what our docs say and that provides us with a lot of guidance but then when different attending's are kind of providing different guidance, it just, you know, added some more confusion to the mix." – EM Nurse

"...my automatic is to go to the charge nurse and be like, what is happening today, please tell me? And then I would trust and her and just like, go with it." – EM Resident

Education is integral

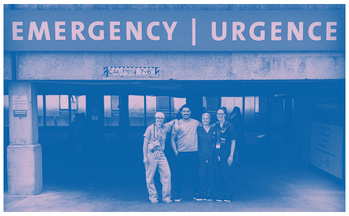

There is perhaps no greater evidence that education is integral to the practice of emergency medicine than the emergence of a novel viral pandemic as an existential threat. In a matter of weeks COVID-19 went from a far-off threat to a very present potential reality. There was a palpable worry around the changes in protocol, operations, and the disease itself.

“Any fine motor skill, if you switch it up in the least, it's uh, or the thinking around it, it can hold you up when there's anxiety involved.” – EM Attending

"...Some of the staff have not intubated anybody in years and all of the sudden they're now having to do this high-stakes intubation with everybody looking at them through the glass and told to do it a different way than they normally would.” – EM Attending

In response, the mobilization of education and training within the department was dramatic. Within a matter of weeks all attending physicians were involved in a mandatory simulation-based airway training program. At the same time a daily in-situ walk through/simulation of COVID intubations was rolled out in the department. Both of these initiatives were unprecedented in scope.

“I think the sims, and really what has been a team thing supported by the department and the urgency of the situation has kind of contributed to buy-in, but I think people have valued that and that may be something that we can channel and continue on in the future.” – EM Attending

There were numerous examples of the simulation exercises translating into enhanced real-life team function.

“We had to get ready very quickly because it [the ambulance] was literally 5 minutes out and the group that was there, like we had literally just finished a sim on what we were going to do and so it was like, okay, we all just did this, we're just gonna do it again for real this time and so like, the people in the room were all on the same page....” – EM Nurse

Key Findings

- Education/training **resources and energy can be channeled** in moments of need and will
- **Social and cultural objectives** of learning events are relevant
- The call for clinicians to adapt practice and educate and/or maintain research productivity during crisis is a significant challenge

While the sim sessions were powerful some felt they may have propagated hierarchy and could have been more inclusive.

"I do feel that they've been quite heavy on the physician role and I think that, I do like, definitely we've been incorporating nursing and incorporating RT, but I feel like the focus is always on the airway and always on getting from kind of the non-intubated to the intubated patient and I think that it would be nice to have the opportunity to also kind of explore more, kind of focus on the nursing like, how are the nurses gonna kinda facilitate the safest of doing things" - EM Nurse

Though COVID-19 fueled some aspects of education and training, it presented threats to other domains such as grand rounds, retreats, and many aspects of resident education. Medical students were excluded entirely from care in the hospital.

“The culture of our program I think evolves around us all sitting around in Richardson Lab Amphitheatre on Thursdays and you know, everybody kind of getting together, having their coffee, you know, having a nice presentation and I think it sort of brings people together so that's, that's gonna be a culture change if we are unable to do that in the future, or for a long time.” - EM Attending

“Covid has taxed teaching, not by making the environment difficult to be a good teacher in, I don't think, but instead by putting additional difficulties on cognitive load and personal reserve, which allow for enthusiasm, focus and dedication to being an educator.” – EM Attending

“I do worry about the resident experience throughout all this. I don't think, it doesn't keep me up at night, like losing, being absent from my children as a father does, but I think that I do worry about that this is a major threat to the educational experience.” – EM Attending

The threat of COVID-19 and associated public health realities resulted in educational adaptations including a new video conference-based core rounds and grand rounds. Informal mentorship has also been affected. This educational evolution and negotiation is ongoing.

Emergency medicine is part of self-identity

The realities of the pandemic sometimes aligned and sometimes conflicted with the self-identity of those who work in the emergency department as well as the developing professional identity of those in training. Some valued personality traits of those working in the ED – like adaptability, calmness, friendliness, and adaptability – were protective in the face of COVID-19.

*"I kind of try and keep as **calm** as possible and keep other people calm too you know, because I guess that's just my personality."* – Environmental services

*"I think we're pretty **adaptable** so I think the trainees are pretty adaptable so I think that it will all be okay."* – EM attending

However, many in the circumstances found themselves struggling with feelings that were not in keeping to their usual demeanour which could be a potential threat to identity.

*"I know it's been difficult for everyone, um, but then there's been a lot of sort of um, a bit, sorry, not a lot, a little bit of negativity stemming from that and I think that it has to do with like, and I've sort of been feeling these things as well like, **confusion and anxiety and um, and I'm not an anxious person.**"* – EM Nurse

*"I think that we operate with pattern and the usual song and dance of being an Emergency physician is totally different now and yes, we still go and see a patient, but every step in the process is now changed from how I leave my house, all the way through to what happens when I walk through my door when I get home and so, that clinical element and then **anxiety level at work is heightened and so it's harder to enjoy all of the elements** because what you do is you build your patterns that you just rest on"* – EM Attending

*"You can't just do the things that you got real comfortable doing and then enjoy the features of the job that you really love because they are different now, you know, they are just different. **You can't even enjoy the interpersonal interactions** ."* – EM Attending

"This is a first time in my career that, you know, you thought about there might be involved with something that you could catch that you could die from and, you know, I never, ever think that way." – EM Attending

The intersection of risks of the job with family life and personal identity was sharp during the acute phase of the pandemic with many staff undergoing extensive decontamination protocols and arranging for emergency housing and contingency plans should they become infected.

"Everything that I have with me that needs to go home is Virox cleaned before it goes into a clean part of my bag...anything that I'm wearing goes into a separate bag..." – EM Nurse

"I think it's just an amplified worry that statistically no matter what happens, you know, some of us are going to get this thing and a smaller number are going to get sick and even smaller number could die. It's hard to ignore for sure, so you know, we made sure our wills are up to date and that you know, there is clear instructions for what happens if one of us gets sick" – EM Attending

Perhaps in an effort to combat these potential identity threats the community seemed to rally together.

"I've tried to implement at this point is kind of more one on one and just checking in with the juniors, um and doing that in different ways like, whether it's going for like a socially distanced walk..."- EM Resident

Interestingly Kingston never really having a first wave of COVID posed another type of conflict in identity - some in our community felt underutilized and over celebrated. This conflict in identity likely contributed to the decision for staff physicians' donation to the Lion's Heart Foundation to support the greater Kingston community.

"This might be the defining medical event of our generation and it's kind of sad being an Emergency doctor, like part of me like really wants to be on the frontline or, you know, making a difference instead of actually doing less than I was doing before." - EM resident

"We have been on the receiving end of tremendous community support over the last three months...And yet, we are also very privileged... we recognize that our experience is actually far better than that of many in our community for whom this has truly been a crisis" – Daily Update

Key Findings

- Crisis may result in the manifestation of traits and feelings that are in **conflict with self-identity**
- The physical threat associated with COVID-19 highlights **overlap between aspects of identity** (i.e. home and work)

Belonging to the ED team

Reading this we hope - while recognizing we are far from perfect - that you might feel as proud as we do for being part of this adaptable and principled team.

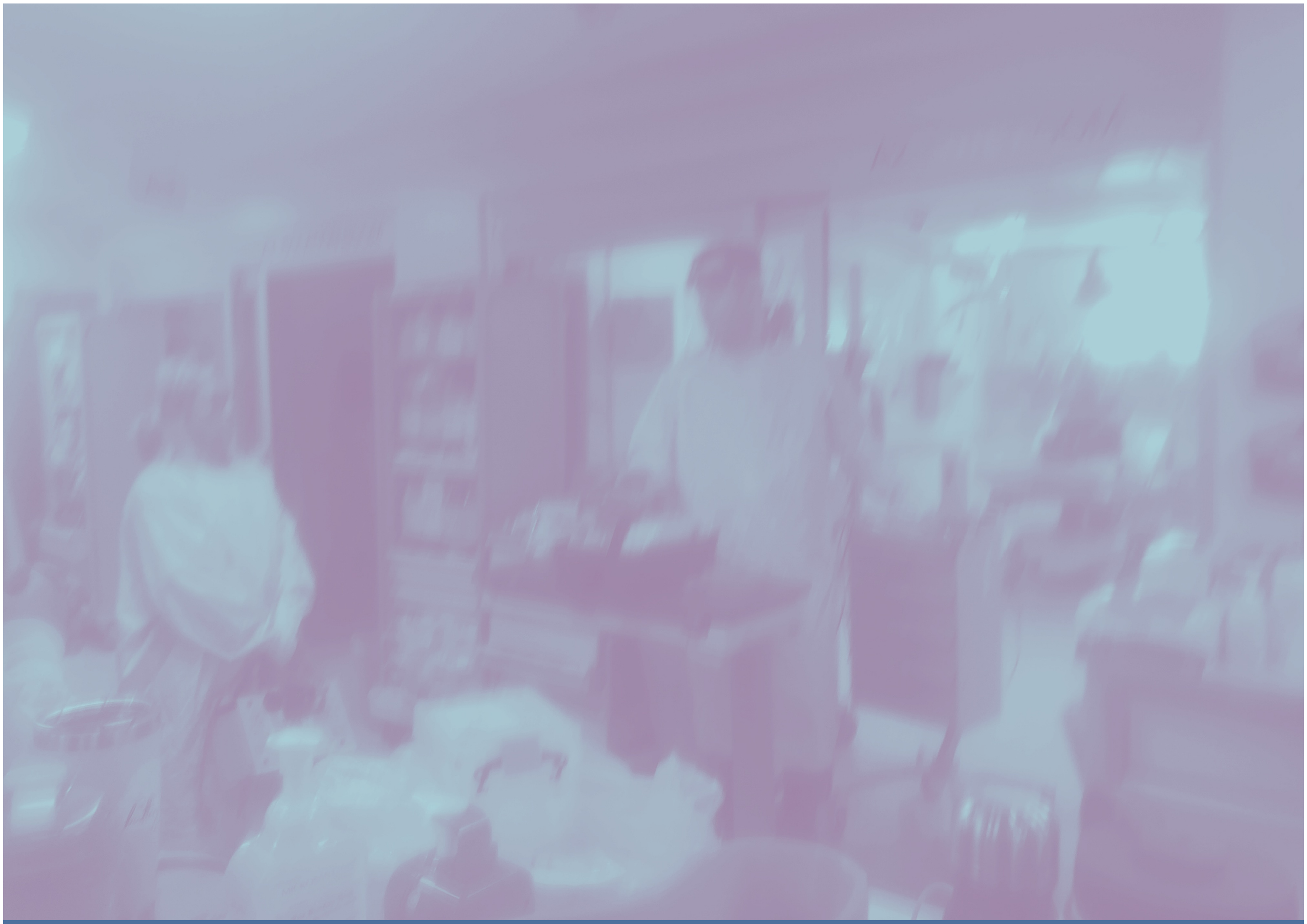

As mentioned in the introduction we look forward to hearing your thoughts about whether this analysis of this unique time resonates What does this mean for you? Stay tuned for an opportunity to discuss virtually or get in touch with Eve Purdy (epurdy@qmed.ca), Stuart Douglas (Stuart.Douglas@queensu.ca) or any member of the team .
